# Molecular surveillance of tuberculosis-causing mycobacteria in wastewater

**DOI:** 10.1016/j.heliyon.2022.e08910

**Published:** 2022-02-04

**Authors:** Hlengiwe N. Mtetwa, Isaac D. Amoah, Sheena Kumari, Faizal Bux, Poovendhree Reddy

**Affiliations:** a Department of Community Health Studies, Faculty of Health Sciences, Durban University of Technology, PO Box 1334, Durban, 4000, South Africa; b Institute for Water and Wastewater Technology (IWWT), Durban University of Technology, PO Box 1334, Durban, 4000, South Africa

**Keywords:** Tuberculosis, Mycobacteria, *Mycobacterium tuberculosis* complex, Wastewater, Droplet digital PCR

## Abstract

The surveillance of tuberculosis infections has largely depended on clinical diagnostics and hospitalization data. The advancement in molecular methods creates an opportunity for the adoption of alternative surveillance systems, such as wastewater-based epidemiology. This study presents the use of conventional and advanced polymerase chain reaction techniques (droplet digital PCR) to determine the occurrence and concentration of total mycobacteria and members of the *Mycobacterium tuberculosis* complex (MTBC) in treated and untreated wastewater. Wastewater samples were taken from three wastewater treatment plants (WWTPs) in the city of Durban, South Africa, known for a high burden of TB/MDR-TB due to HIV infections. All untreated wastewater samples contained total mycobacteria and MTBC at varying percentages per WWTP studied. Other members of the MTBC related to tuberculosis infection in animals, *M. bovis* and *M. caprae* were also detected. The highest median concentration detected in untreated wastewater was up to 4.9 (±0.2) Log10 copies/ml for total mycobacteria, 4.0 (±0.85) Log10 copies/ml for MTBC, 3.9 (±0.54) Log10 copies/ml for *M. tuberculosis*, 2.7 (±0.42) Log10 copies/ml for *M. africanum*, 4.0 (±0.29) Log10 copies/ml for *M. bovis* and 4.5 (±0.52) Log10 copies/ml for *M. caprae*. Lower concentrations were detected in the treated wastewater, with a statistically significant difference (P-value ≤ 0.05) in concentrations observed. The log reduction achieved for these bacteria in the respective WWTPs was not statistically different, indicating that the treatment configuration did not have an impact on their removal. The detection of *M. africanum* in wastewater from South Africa shows that it is possible that some of the TB infections in the community could be caused by this mycobacterium. This study, therefore, highlights the potential of wastewater-based epidemiology for monitoring tuberculosis infections.

## 1 Introduction

Tuberculosis (TB) is a notifiable communicable disease caused by a group of closely related, slow-growing mycobacteria collectively named *Mycobacterium tuberculosis* complex (MTBC) (Forbes et al., 2018). Most TB infections in humans are caused by *M. tuberculosis,* however, there are other members of the MTBC that cause TB in both humans and animals. These include *M. bovis*, the causative agent of tuberculosis mainly in animals.*M. bovis* causes disease in humans because of its zoonotic capacity, but it is mainly the agent of animal and particularly bovine TB infections (bovines being the main maintenance host of the bacterium) (Walter et al., 2012; Howell et al., 2019; Nugent et al., 2017; Inlamea et al., 2020), *Mycobacterium africanum*, the causative agent of human tuberculosis (mainly in Western Africa) (Gehre et al., 2016; Tientcheu et al., 2016) and *Mycobacterium canetti* isolated in the Horn of Africa (Loukil et al., 2019) and reported in human infections, however, its natural reservoir, host range, and mode of transmission still remains debatable. Other members of this group are *M. microti*, *M. caprae, M. pinnipedii* and *M. mungi,* usually associated with animal infections with possible transmission to humans.

In 2019, an estimated 10.0 million (range, 8.9–11.0 million) people worldwide were infected with tuberculosis, with 1.5 million deaths per year (Visca et al., 2021; World Health Organization, 2020a, World Health Organization, 2020b). In most African countries, particularly in South Africa, HIV infection is regarded as a significant risk factor for contracting tuberculosis (TB), with co-infection linked to increased morbidity and mortality (Mesfin et al., 2014; Travis et al., 2019). Over 70% of people with TB and HIV/AIDS (6 million) live in Sub-Saharan Africa, where bovine TB is a threat to human health (Ayele et al., 2004, Bovine Tuberculosis, 2021; Ntloko et al., 2019). WHO produces annual estimates of the burden of disease caused by tuberculosis, which are measured in terms of incidence, prevalence, and mortality, based on data collected through surveillance systems (case notifications and death registrations), special studies (including prevalence surveys), mortality surveys, "inventory studies" of under-reporting of detected TB, in-depth analysis of surveillance and other data, expert opinion, and consultations with countries (Glaziou et al., 2016). However, in resource-poor countries, monitoring of tuberculosis/drug-resistant tuberculosis (DR-TB) is a major challenge because assays are costly and time-consuming, and laboratories are ill-equipped. This has led to underestimation/reporting of TB cases in such countries (Oga-Omenka et al., 2020; Zarowsky et al., 2020; World Health Organization, 2020a, World Health Organization, 2020b), therefore alternative means of estimating to complement the existing surveillance systems would be beneficial. There is evidence to support the detection of tuberculosis causing organisms in human faeces (Dürr et al., 2013; Bosch, 2018; Abaye et al., 2017; Walters et al., 2018). Few studies from the 1960s–70s reported on the isolation of *M. tuberculosis* from the environment, such as hospital sewage (Buczowska, 1965; Buraczewski and Osinski, 1966), households (Buczowska, 1965; Buraczewski and Osinski, 1966; Poptsova, 1974) and farms (Kazda et al., 2010; Skurski et al., 1965; Szulga et al., 1965). Therefore, the concept of wastewater-based epidemiology (WBE) could be adopted to provide additional information on the TB burden. WBE assumes that any stable substance excreted by humans and found in sewage/wastewater can be used to estimate the original concentration excreted by the serviced population. When pathogens are excreted in the feces/urine of infected people, the same concept can be used to analyze pathogen circulation in sanitary sewers in a given population (Prado et al., 2021; Mao et al., 2020; Polo et al., 2020). This method is useful, especially when clinical diagnosis resources are limited and reporting systems are unavailable or inefficient (Kitajima et al., 2020; Hart and Halden, 2020; Thompson, 2020; Prado et al., 2021). This approach has seen increased interest during the COVID-19 pandemic (Ahmed et al., 2020; Gonzalez et al., 2020; Hart and Halden, 2020) WBE studies may be able to aid in the reconstruction of spatially explicit transmission chains, such as not only "who infected who," but also "where they were infected," which may provide insight into how they were infected (Sims and Kasprzyk-Hordern., 2020; Martinez et al., 2019).

TB primarily spreads person-to-person by aerosolized infective tubercle particles (Shiloh, 2016). However, there are observations that TB could be transmitted through other means, such as through faecal-oral transmission (Santos et al., 2015; Lombard, 2011; Allen et al., 2021). Despite the potential role of the environment in TB transmission, there is limited information on the occurrence of the causative organisms in the environment. This could be attributed to the lack of sensitive and scalable techniques to detect MTBC in environmental samples (Santos et al., 2015; Barbier et al., 2016; Martinez et al., 2019). *M. tuberculosis* can be cultured from soil and other materials, but due to bacterial overgrowth and the presence of "differentially culturable" (or "viable but non-culturable") organisms, sensitivity may be limited (Martinez et al., 2019; Chengalroyen et al., 2016; Mukamolova et al., 2010; Velayati et al., 2015). Methods for optimal promotion of *M. tuberculosis* complex's growth following recovery from the environment are needed to gain a better understanding of their viability in various environmental matrices (Martinez et al., 2019). This challenge could be addressed with the use of molecular techniques, such as the reverse transcription polymerase chain reaction (RT-PCR) (Lu et al., 2003; Young et al., 2005). Molecular detection of *M. tuberculosis* complex has been demonstrated in filtered air samples (Wood et al., 2016), but few studies are investigating its detection in water or wastewater samples (Fine et al., 2011; Velayati et al., 2015; Li et al., 2015; Rosso et al., 2018; Guo et al., 2019).

The use of more sensitive and accurate molecular methods for the detection of tuberculosis-causing mycobacteria in wastewater could play a significant role in developing the WBE approach for estimating the TB burden. This will complement or be used as an alternative to the current surveillance methods in place. Additionally, these methods could theoretically be used to ascertain the potential risk of TB infection in community settings due to exposure to wastewater. The aim of the paper is to evaluate a molecular surveillance strategy for the detection of tuberculosis-causing mycobacteria in both untreated and treated (post-chlorination) wastewater in KwaZulu Natal (KZN), South Africa.

## 2 Methodology

### 2.1 Study site

Three wastewater treatment plants (WWTPs) in the city of Durban, South Africa, were sampled for municipal wastewater: WWTP A, WWTP B, and WWTP C, on four different occasions. The WWTPs were chosen based on whether or not they served a population of at least 10,000 people and whether or not they received hospital sewage. The treatment configurations and capacities of these WWTPs were also different. For example, WWTP A does not have an activated sludge treatment or secondary clarification process, whereas WWTP B and C both do. There are other differences in the treatment processes, as shown in Table 1 and the schematic diagrams in Figures S1-S3 (Supplementary material), that are important to consider when discussing the efficacy of these WWTPs in removing mycobacteria.

**Table 1:** Details of the wastewater treatment plants used for this study.

Treatment works	Design Capacity (Mℓ/d)	Primary Settling	Activated Sludge	Secondary clarification	Bio-filters	Sludge Digestion	Tertiary treatment	Remarks
WWTP A	18.8	Yes	No	No	Yes	Yes	Yes	Receives from **Hospital A**, which has 17 clinics and provides health care to the community on a regional and district level, receives funds. The hospital is one of the sites for HIV transmission from mother to child (MTCT) and is home to the country's largest crisis center, now known as the 'Place of Comfort.'
WWTP B	4.90	N/A	Extended Aeration	Yes	No	No	Yes	Receives from **Hospital B,** which serves the people of Chatsworth and the surrounding area, as well as the Inner and Outer West, with boundaries extending from Yellowwood Park to Richmond. This hospital also serves as a referral center for another hospital and clinic.
WWTP C	70.0	Yes	Conventional	Yes	No	Yes	Yes	Receives from **Hospital C** complex which offers specialised services for **Multi Drug Resistant (MDR) and complicated TB**

Information sourced from: Cross and Buckley (2016); Mtetwa et al. (2021).

### 2.2 Sample collection and processing

Each WWTP's influent (raw/untreated wastewater) and effluent (treated wastewater) received a 1-L composite sample. Composite samples made up of many subsamples were taken, for example, one small sample was taken, followed by a 30-second interval, and then the next sample was taken, and so on, until the required sample volume (1 L) was reached. The samples were transported to the lab in an ice-filled cooler box, kept at 4 °C, and analyzed within 48 h. Per WWTP, two samples were taken (influent and effluent (post-chlorination)). Samples were homogenized before analysis, and 50 mL subsamples were taken and centrifuged at 3000 rpm for 20 min, with the supernatant discarded and the pellet used for DNA extraction. The DNA was extracted using a DNeasy Powersoil DNA extraction kit (QIAGEN) according to the manufacturer's instructions, with no changes. IMPLEN NanoPhotometer NP80 – All-in-One Spectrophotometer was used to determine the quantity and quality of the extracted DNA. All of the analyses were carried out in triplicate.

### 2.3 Optimization of polymerase chain reaction (PCR) conditions for detection of target organisms in wastewater

Method optimization was done using published primers targeting total mycobacteria, *M. tuberculosis* complex*, M. tuberculosis*, *M. africanum, M. bovis* and *M. caprae*. In this study, regions of differences (RDs) in these various organisms were targeted based on their uniqueness. However, it must be noted that some of these regions of differences are shared among the species within the *M. tuberculosis* complex and difference studies report them differently. For instance, the total mycobacteria were targeted using the 16s rRNA gene (Chae et al., 2017), Rv0577 for *M. tuberculosis* complex (Chae et al., 2017), RD9 for *M. tuberculosis* (Perez-Osorio et al., 2012; Chae et al., 2017), RD1 present for *M. bovis* (Kim et al., 2013), RD4 present for *M. caprae* (Domogalla et al., 2013). During the method development and optimization, the PCR amplicons were sent for sequencing for confirmation of the organisms targeted.

The limit of detection (LOD) of the PCR protocol was determined using positive control DNA of *M. tuberculosis* H37Rv strain. The concentration of the target was diluted (10^−1^- 10^−4^) to the following copies/μl: 59.2, 18.6, 13.75 and 7.06 respectively for conventional PCR to determine the lowest concentration of *M. tuberculosis* detectable.

The lowest detectable concentration of *M. tuberculosis*. The PCR mixture for all targeted organisms contained 12.5 ul of OneTaq 2X Master Mix with Standard Buffer (New England BioLabs inc), 2 l of primer mix (final concentration of 0.2–0.4 M), 1 l (60 ng/l) of DNA template, and 9.5 l of molecular grade water in a final volume of 25 l in a reaction tube. The VeritiTM 96-Well Thermal Cycler was used for PCR amplification. Optimised thermocycling conditions were initial denaturation step at 95 °C for 10 min and followed by 30 cycles of 96 °C for 45 s. The annealing temperatures varied for each primer (organisms), total mycobacterial species (16s rRNA gene- 500bp) at 61.5 °C for 45 s, *M. tuberculosis* complex (Rv0577-700bp) at 54 °C for 60 s, *M. tuberculosis* (RD9 present- 369 bp) at 59 °C for 60s*, M. africanum* (RD8 present- 150 bp) at 68 °C for 60 s, *M. bovis* (RD1 present-264bp) at 57 °C for 60s and *M. caprae* (RD4 present) at 58 °C for 60s and extension at 72 °C for 40 s. The final extension step was performed at 72 °C for 10 min.

After amplification, 3 μl of ethidium bromide (final concentration of 0.2–0.5 μg/mL) was added on 2% agarose gel in of 1× Tris-borate-EDTA (TBE) buffer and analysed via agarose gel electrophoresis. The assay products were electrophoresed for 45–60 min at 70 V in 1× TBE buffer, and the gels were visualized using a Bio-Rad Gel Doc™ XR.

### 2.4 Determination of the presence of total mycobacteria, MTBC, *M. tuberculosis, M. africanum*, *M. bovis and M. caprae* in treated and untreated wastewater by conventional PCR

The optimized conventional PCR protocol was used to determine the presence of the selected organisms or group of organisms in wastewater from Durban, South Africa. Wastewater samples were collected and processed using the methodology described above (Section 2.2).

### 2.5 Determination of the concentration of total mycobacteria, MTBC, *M. tuberculosis, M. africanum*, *M. bovis and M. caprae* in treated and untreated wastewater

The Rv0577 primer for *M. tuberculosis* complex mentioned in section 2.3 was used to determine the limit of detection for *M. tuberculosis* using a measured DNA template (1.5 ng/μl of *M. tuberculosis* H37Rv strain). The DNA template was serially diluted from 10^−1^ to 10^−9^.

The concentration of these organisms was determined using the droplet digital PCR (ddPCR). The same set of primers presented in Table 2 were used. The ddPCR analysis was performed in a 20 μL reaction volume, containing, 10 μL of 2X QX200 ddPCR EvaGreen Supermix (Bio-Rad), 1–20 ng/μL of template DNA quantified by the IMPLEN NanoPhotometer NP80 – All-in-One Spectrophotometer, forward primers (FP) and reverse primers (RP), each at a final concentration of 250 nM and RNase/DNase free water.

**Table 2:** Median (standard deviation) concentration and range of the concentration (Log10 copies/mL) of total Mycobacteria, *M. tuberculosis* Complex, *M. tuberculosis, M. africanum, M. bovis and M. caprae* in influent and effluent wastewater at the three WWTPs.

	WWTP A				WWTP B				WWTP C			
	Influent		Effluent		Influent		Effluent		Influent		Effluent	
	Median(±SD∗)	Range	Median(±SD)	Range	Median(±SD)	Range	Median(±SD)	Range	Median(±SD)	Range	Median(±SD)	Range
Total mycobacteria	4.9 (0.2)	4.8–5.2	4.4 (0.03)	4.3–4.4	4.8 (0.06)	4.8–4.9	3.9 (0.15)	3.8–4.2	4.8 (0.47)	4.4–5.2	4.0 (0.8)	3.2–4.7
MTBC#	4.0 (0.85)	2.3–4.2	2.9 (0.94)	1.8–3.9	3.8 (0.33)	3.2–4.0	3.4 (0.52)	2.8–4.0	3.3 (0.52)	2.5–3.8	3.0 (0.44)	2.5–3.4
*M. tuberculosis*	3.9 (0.31)	3.3–4.0	3.5 (0.34)	3.3–4.1	3.9 (0.54)	2.9–4.1	3.7 (0.44)	3.2–4.1	2.8 (0.75)	2.8–4.3	2.7 (0.62)	2.5–3.8
*M. africanum*	2.3 (0.29)	2.0–2.7	2.5 (0.31)	2.0–2.7	2.5 (0.29)	2.2–2.8	2.3 (0.18)	2.2–2.6	2.7 (0.42)	2.1–3.1	2.5 (0.16)	2.3–2.6
*M. bovis*	4.0 (0.29)	3.5–4.2	2.8 (0.97)	2.0–4.1	3.8 (0.43)	3.4–4.2	3.8 (0.74)	2.5–4.1	3.6 (0.44)	3.2–4.2	3.9 (0.68)	3.2–4.5
*M. caprae*	4.0 (0.36)	3.7–4.5	3.7 (0.36)	3.2–4.1	4.5 (0.52)	3.5–4.7	4.3 (0.87)	2.7–4.6	4.1 (0.81)	2.5–4.3	3.8 (0.84)	2.7–4.6

∗ Means Standard deviation.

# refers to *M. tuberculosis* complex, n = 4.

Droplets were generated using the automated droplet generator and the following amplification protocol was followed: Optimised thermocycling condition included an initial denaturation step at 95 °C for 10 min and followed by 30 cycles of 96 °C for 45 s. The annealing temperatures varied for each primer (organisms), total mycobacterial species (16s rRNA gene- 500bp) at 61.5 °C for 45 s, *M. tuberculosis* complex (Rv0577-700bp) at 54 °C for 60 s, *M. tuberculosis* (RD9 present- 369 bp) at 59 °C for 60s*, M. africanum* (RD8 present- 150 bp) at 68 °C for 60 s, *M. bovis* (RD1 present-264bp) at 57 °C for 60s and *M. caprae* (RD4 present) at 58 °C for 60s and final incubation step was performed at 98 °C for 10 min (ramp rate 2.2 °C/s). These conditions were applied to the wastewater samples. After thermal cycling, the ddPCR plates were read using the QX200 droplet reader (Bio-Rad). Droplet counts and amplitudes were analysed with QuantaSoft™ analysis Pro software (Bio-Rad).

The standard/reference *M. tuberculosis* (H37Rv strain) DNA was determined to have an average of 9226 (±642.1) copies/mL using this protocol/assay. The LOD after ten-fold serial dilutions was determined to be 3.0 (±0.06) gc/ml (Figure S4) with an average of 18,892 droplets generated per well.

### 2.6 Statistical analysis

Descriptive statistics was calculated using Microsoft Excel, and a test of normality was conducted using the Akaike Information Criterion (AIC) score, which was calculated using @Risk (Palisade Inc. USA). The Kruskal-Wallis tests, followed by Dunn's Multiple Comparison tests, were used to compare the concentrations of different tuberculosis-causing mycobacteria based on the normality tests. The Mann-Whitney tests were used to compare the concentrations of mycobacteria in untreated and treated wastewater. All statistical tests had a 95% confidence interval, and a p-value of less than 0.05 was considered statistically significant. GraphPad Prism was used for all statistical analyses (Version 7.0, GraphPad Software, USA).

## 3 Results

### 3.1 Determination of the presence of total mycobacteria, MTBC, *M. tuberculosis, M. africanum*, *M. bovis and M. caprae* in treated and untreated wastewater by conventional PCR

**Figure 1:**
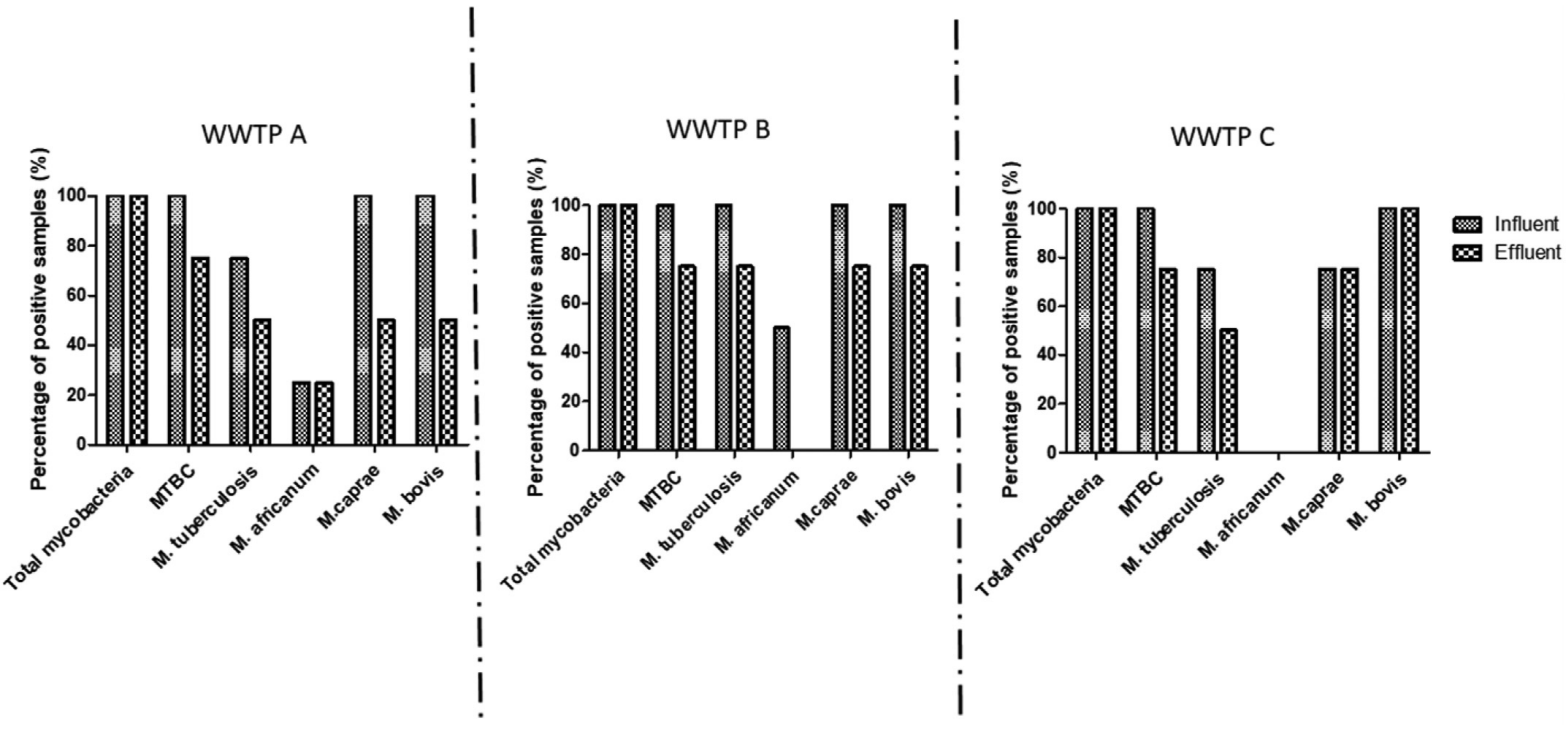
Percentage of influent and effluent wastewater samples showed positive for total mycobacteria, *M. tuberculosis* Complex, *M. tuberculosis, M. africanum, M. bovis* and *M. caprae* (N = 4).

Conventional PCR was optimized with limit of detection determined to be 18.6 copies/μl using Rv0577 for total *M. tuberculosis* complex primer. The detected mycobacterial organisms varied between the three WWTPs. Total mycobacteria was detected in all (treated and untreated) wastewater samples analysed (Figure 1). Similarly, *M. tuberculosis* complex (MTBC) was present in all (100%) untreated and majority (75%) of treated wastewater samples from all the three WWTPs (Figure 1). *M. bovis* and *M. caprae* were detected in 100% of all untreated wastewater and *M. bovis* was present in 50%, 75% and 100% of the treated wastewater in WWTP A, WWTP B and WWTP C respectively. *M. tuberculosis*, the main causative agent for human tuberculosis, was detected in 75% of untreated samples from both WWTP A and WWTP C and 100% of untreated wastewater samples from WWTP B.

The least prevalent was *M. africanum*, which was detected in 25% of untreated samples in WWTP A, 50% in WWTP B, and not detected in WWTP C. However, their presence in the treated wastewater was lower, with 25% at WWTP A and no detection in both WWTP B and WWTP C.

### 3.2 Determination of the concentration of total mycobacteria, MTBC, *M. tuberculosis* spp., *M. africanum* spp., *M. bovis* spp., *M. caprae* spp. in treated and untreated wastewater

Comparing the concentration of the six organisms or group of organisms measured in the untreated wastewater across the three WWTPs showed differences in their concentrations. Between the WWTPs, a statistically significant difference in concentrations of each organism was observed. The highest median concentration for total mycobacteria in the untreated wastewater was recorded in WWTP A (4.9 (±0.2) Log10 copies/ml), with the lowest median concentration of 4.8 (±0.06) and 4.8 (±0.47) Log10 copies/ml in WWTP B and WWTP C, respectively. The *M. tuberculosis* complex members were more abundant in WWTP A, with a concentration of 4.0 (±0.85) Log10 copies/ml as compared to both WWTP B (3.8 (±0.33) Log10 copies/ml) and WWTP C (3.3 (±0.52) Log10 copies/ml).

WWTP A and WWTP B influent had similar median concentrations of *M. tuberculosis* (Table 2) and lower concentration of 2.8 (±0.75) Log10 copies/ml was recorded in WWTP C. The organism with the lowest concentrations irrespective of WWTP was *M. africanum,* this correlated with the percentage of samples with positive detection of this organism described in the section 3.1 above (Figure 1). As shown in Table 2, the other two rarely occurring mycobacteria (*M. bovis* and *M. caprae*) were also recorded in low concentrations. However, comparing with *M. bovis* and *M. caprae,* the concentrations of *M. africanum* in the untreated wastewater samples were significantly lower (P value ≤ 0.05).

Furthermore, within each WWTP, the concentration of these organisms was lower in the treated wastewater compared to the untreated wastewater concentrations described above. For instance, the concentration of total mycobacteria in the treated and untreated wastewater were statistically significant (P value ≤0.05) in all the WWTPs, except WWTP C. However, the difference in MTBC, *M. tuberculosis, M. africanum, M. bovis* and *M. caprae* concentrations in the treated and untreated wastewater in all the WWTPs was not statistically significant (P value ≥0.05). Despite the reductions observed in all the WWTPs, concentrations up to 4log10 for these mycobacteria are released into receiving water environments (Table 2).

### 3.3 Reduction in the concentration of tuberculosis-causing mycobacteria during wastewater treatment

**Figure 2:**
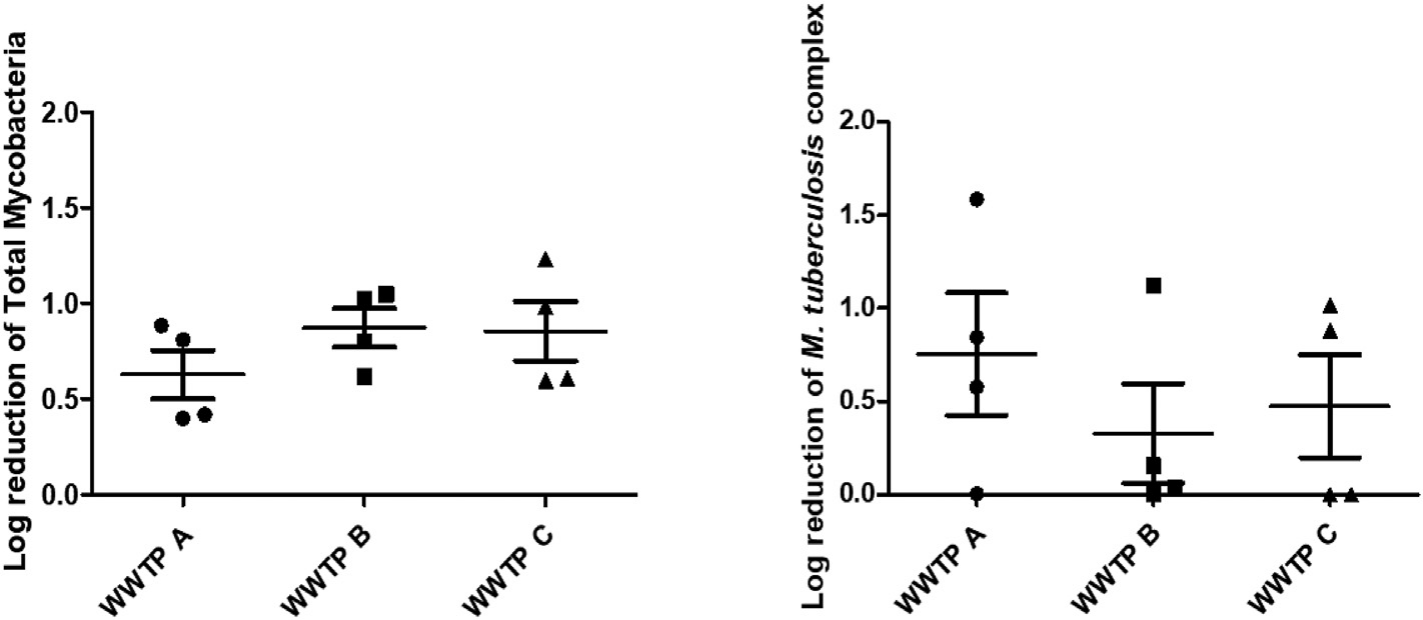
Log reduction of total Mycobacteria and *M. tuberculosis* complex achieved by each WWTP.

**Figure 3:**
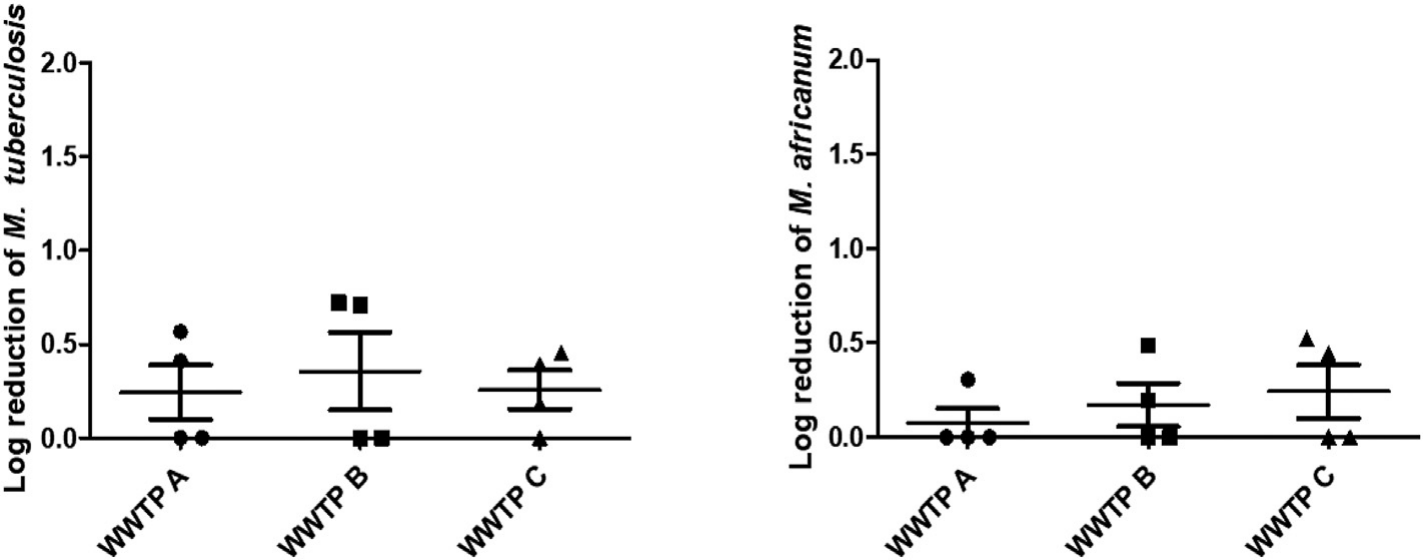
Log reduction of *M. tuberculosis* and *M. africanum* achieved by each WWTP.

**Figure 4:**
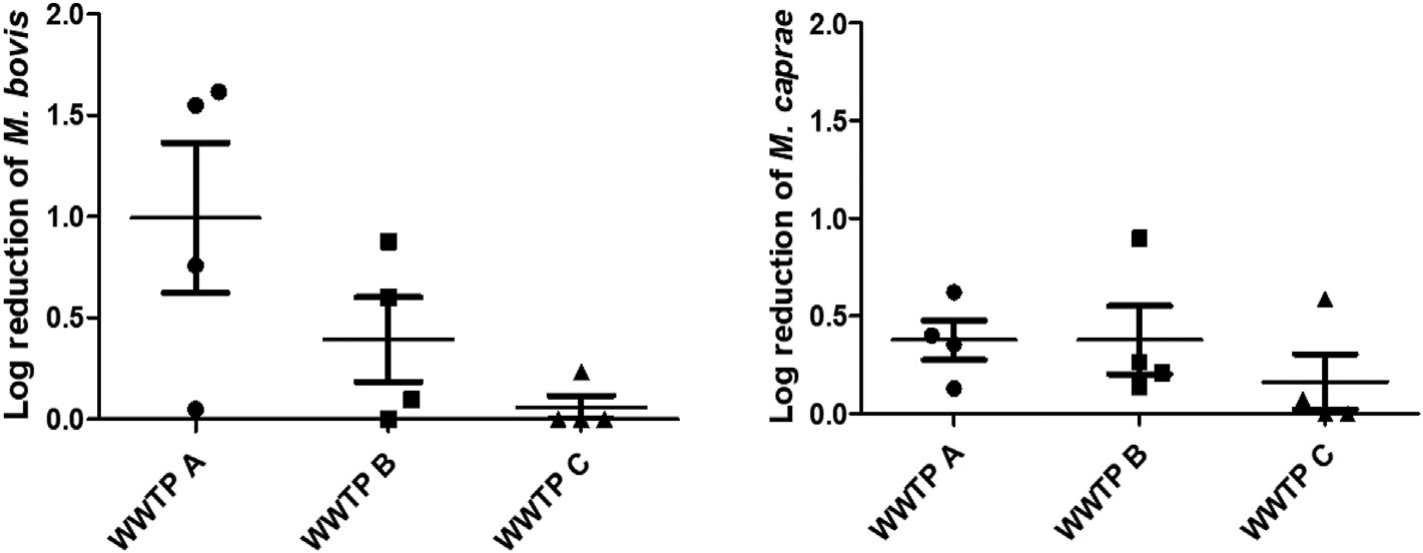
Log reduction of *M. bovis* and *M. caprae* achieved by each WWTP.

The observed log reduction in each WWTP as presented in Figures 2, 3, and 4 did not show any statistically significant differences when compared between the three WWTPs, irrespective of the organism or group of organisms (P value ≥0.05). The highest median log reduction of 0.91 (±0.20) for total mycobacteria was achieved by WWTP B. Similarly, WWTP A, had the highest median log reduction in *M. tuberculosis* complex members of 0.71 (±0.65). Despite these differences in log reduction as can be seen in Figure 2, the Kruskal-Wallis test did not show any statistically significant differences.

Specifically, looking at tuberculosis causing mycobacteria, the highest median reduction in *M. tuberculosis* was observed in WWTP B (0.36 (±0.41)) and the lowest at WWTP A (0.21 (±0.29)). In respect of *M. africanum*, the highest reduction was recorded at WWTP C (0.22 (±0.28)) and the lowest at WWTP A (see Figure 3). The highest log reduction of *M. bovis* and *M. caprae* was observed in WWTP A (Figure 4). This study could not identify any single WWTP to have the most efficient log reduction for all mycobacteria tested.

## 4 Discussion

Wastewater contamination with total mycobacteria, members of the *M. tuberculosis* complex and the other tuberculosis-causing mycobacteria could be attributed to several factors, including shedding of these organisms in human and animal faeces which end up in wastewater treatment plants from hospital sewage or domestic sewage. This study has shown the presence of different species of *Mycobacterium* in SA wastewater in varying abundance. The presence of mycobacteria known to cause tuberculosis infections in humans was considerably lower than the total mycobacteria, which is expected as it is comprising of all species of *Mycobacterium*. The prevalence of *M. tuberculosis* in untreated wastewater was between 75%-100% and *M. africanum* ranged from 25%-50% (Figure 1). Comparing the two species, the prevalence of *M. tuberculosis* in this study correlates with the high prevalence reported in clinical studies conducted in KZN (Mzembe, 2020; Naidoo et al., 2018; Brown et al., 2019). *M. caprae* and *M. bovis* was prevalent in 100% of the untreated wastewater from WWTP A and WWTP B and 75% of untreated wastewater for WWTP C, for *M. bovis*. Although *M. bovis* and *M. caprae* are mainly known as causative agents of TB in animals, reports of human TB caused by these mycobacteria have been published (Prodinger et al., 2014; Lan et al., 2016). Bhembe, and Green (2020) reported via molecular analysis of sputum samples that 8.7% of 184 TB patients were infected with *M. caprae.* Therefore, the presence of this mycobacterium in the wastewater could be due to the excretion in feces of both infected humans and animals. One of the significant findings is the detection of *M. africanum* in wastewater within South Africa. This organism consists of two phylogenetically distinct lineages, the *M. africanum* West African 1 (MAF1) and *M. africanum* West African 2 (MAF2) (Gagneux et al., 2006). *M. africanum* is endemic to West Africa and is known to cause up to half of the human TB in that region (De Jong et al., 2010). Therefore, the detection of *M. africanum* in wastewater from South Africa potentially indicates that some of the TB infections within the country could be caused by *M. africanum*. A study in the mid-2000s reported *M. africanum* as not the major cause of tuberculosis in Cape Town, South Africa (Demers et al., 2010), which is supported by the findings in the Eastern Cape province of South Africa, where about 2.2% of TB infections were determined to be caused by *M. africanum* (Bhembe, and Green, 2020). The detection of these organisms could be due to the migration of people from West African countries into South Africa, thereby leading to the potential spread of the mycobacteria within the South African population. The detection of the various tuberculosis-causing mycobacteria, most especially *M. africanum*, in the untreated wastewater shows the potential for molecular surveillance of these organisms in wastewater contributing to WBE. Lorenzo and Picó (2019) proposed that this approach can become an early warning system for outbreaks of disease and a unique tool for the identification of hotspots for pandemics. The usefulness of WBE has already been exhibited with the current studies in relation to COVID-19 infections, where several countries, such Australia (Ahmed et al., 2020), the Netherlands (Naughton et al., 2021), USA (Gonzalez et al., 2020), France (Barcelo, 2020) and South Africa (Pillay et al., 2021) have established national WBE systems. Therefore, the results obtained in this study further advocates this approach in complementing the existing surveillance systems for TB infections.

The concentration of the mycobacteria analyzed varied both by WWTP and by organism. However, it was observed that total mycobacteria and MTBC concentrations were largely within the 4-log_10_ concentrations per ml of wastewater. Comparatively, the concentrations recorded in our study for total mycobacteria or MTBC were higher than the results published by Radomski et al. (2011). In the referenced study, mycobacteria concentrations of up to 2.7 (±2.6) log_10_ copies/mL in untreated wastewater were reported. However, it is worth mentioning that Radomski et al. (2011) focused on non-tuberculosis mycobacteria (NTM). These are also known as environmental mycobacteria, consisting of more than 150 species, and are globally ubiquitous in both natural and man-made environments (Nishiuchi et al., 2017; Tortoli, 2014; Cai and Zhang, 2013, 2014). The higher concentrations determined in this study could potentially be attributed to higher infection numbers in connected populations. The high concentrations observed in WWTP A for all species, could be attributed to the hospital sewage that the WWTP receives. WWTP A receives wastewater from 17 clinics which could represent a highly concentrated sewage compared to the other two WWTPs, regardless of the volume received daily.

The high concentration of the mycobacteria in the treated wastewater (up to 4 log10) could be due to resistant nature of these organisms to environmental conditions and predators. For example, tuberculosis-causing bacteria have been reported to be amoeba-resistant which may enhance their survival in the environment, especially wastewater (Ghodbane and Drancourt, 2013). *M. tuberculosis* and *M. bovis* could survive for hours to days in the amoebal trophozoites (Hagedorn et al., 2009; Mardare et al., 2013). The finding that *M. tuberculosis* and *M. bovis* organisms were engulfed by *Acanthamoeba polyphaga* trophozoites was consistent with previous findings made when *M. tuberculosis* organisms were co-cultured with the free-living amoeba Dictyostelium discodium (Medie et al., 2011; Butler et al., 2020). Additionally, the higher concentrations observed in this could be due to the use of ddPCR platform for quantification of these microbes as against the qualitative PCR (qPCR) technique used by Radomski et al. (2011). The ddPCR platform has been reported to be more sensitive, accurate and less affected by PCR inhibitors, compared to qPCR (Rački et al., 2014; Jahne et al., 2020). Furthermore, it is worth noting that detection of these organisms via PCR does not indicate viability. Therefore, the concentrations detected could be from both live and dead mycobacteria.

The percentage of treated wastewater samples with these organisms was lower than the untreated samples (Figure 1). This could be attributed to the reduction achieved by the wastewater treatment processes. The log reductions of the mycobacteria achieved by the three WWTPs varied, perhaps due to differences in treatment configuration or performance. However, it was observed that each WWTP achieved highest removal for at least one member of MTBC. For instance, the highest log removal for total mycobacteria was WWTP C, WWTP A achieved the highest removal of MTBC and WWTP B had the highest removal of *M. tuberculosis*. These WWTPs have different treatment processes or configurations, for instance WWTP A has four processes i.e primary settling, biofilters, sludge digestion and tertiary treatment (Table 1). In contrast WWTP B has three processes: extended aeration, secondary clarification, and a tertiary treatment process, usually involving chlorination. Therefore, the WWTPs had different treatment processes/steps. This indicates that despite differences in the treatment configuration for these WWTPs, there was no difference in their effectiveness in removing these mycobacteria. Therefore, the removal of mycobacteria could potentially be due to other factors apart from the WWTP configuration. These factors could include the attachment of the mycobacteria cells to solids in the wastewater and capacity of these cells to form biofilms due to the hydrophobic nature of these organisms (Loret, and Dumoutier, 2019; Cao et al., 2019; Jing et al., 2018). Additionally, the operational conditions of the WWTPs could have influenced the reduction achieved. For instance, sub-optimal performance of the WWTPs could potentially result in a less efficient treatment process, thereby resulting the detection of the bacteria in the treated wastewater. Hruska and Kaevska (2012) observed that as of 2012 there were no internationally accepted legal directives on how to control the public health risk associated with environmental mycobacteria. Therefore, the detection of potentially human pathogenic mycobacteria, like *M. tuberculosis* and *M. africanum* and potentially zoonotic species like *M. bovis* and *M. caprae* in the treated wastewater could potentially cause public health issues. However, it must be noted that detection of these organisms via DNA-based PCR does not necessarily indicate the presence of infectious pathogens. Furthermore, it is worth noting that the presence of extracellular DNA in wastewater has been reported (Yuan et al., 2019; Zhang et al., 2019; Slipko et al., 2019; Calderón-Franco et al., 2021). These extracellular DNA could have also contributed to the concentration of the mycobacteria in the treated wastewater. However, several studies have reported the deactivation or removal of extracellular DNA using different methods, such as chlorination, UV (Zhang et al., 2019; Augsburger et al., 2019). Therefore, the concentrations reported in the effluents could be from dead mycobacteria or extracellular DNA, however it is important to pay attention to their presence in the final effluents since a proportion of the concentrations reported could still be viable, posing significant health risks.

### 4.1 Limitations of the study and remarks on further work

Although this study detected members of MTBC in both untreated and treated wastewater, the presence of these organisms may not necessarily translate to tuberculosis infections. Studies on the viability and infectivity of these organisms isolated from wastewater are essential. Also, longitudinal studies to assess the presence of these organisms over a period time are warranted. The detection of these organisms in higher concentration in both untreated and treated wastewater does highlight the need for further studies on the possibilities of health implication from the exposure to untreated wastewater and surface water that may be contaminated with wastewater.

## 5 Conclusion and recommendations

This study was successful in the application of molecular techniques for the detection of total mycobacteria, members of *M. tuberculosis* complex in total, *M. tuberculosis*, *M. africanum*, *M. bovis* and *M. caprae* in untreated and treated wastewater. Detection of these tuberculosis-causing mycobacteria in wastewater could potentially provide insight into infection epidemiology in the connected sewershed, provide information on potential infection risks and help in assessing the efficiency of wastewater treatment plants in removing these organisms. The detection of *M. africanum* in wastewater within South Africa shows the likelihood that some of the TB infections reported in the region could be caused by this bacterium, which is largely reported to be endemic in Western African countries. Furthermore, the detection of *M. caprae* indicates potential zoonotic infections with this mycobacterium as has been reported in some clinical studies. The findings, therefore, make a significant contribution towards the adoption of wastewater-based epidemiology as a cost-effective tool for TB surveillance.

It was also observed that the reduction in mycobacteria concentrations in wastewater could be due to other factors apart from the WWTP configuration; this is based on the observation that the reduction achieved by the different WWTPs was not statistically significant. For instance, the WWTP operational parameters could have potentially impacted on the log reductions observed. Additionally, each WWTP reported highest log reductions for at least one mycobacteria. The detection of potentially human pathogenic species of mycobacteria, such as *M. tuberculosis* and *M. africanum* highlights the potential health risks for populations that may be exposed to either the treated or the untreated wastewater. However, it must be mentioned that PCR data does not differentiate between viable or non-viable bacteria, therefore future studies should focus on determining the viability and infectivity of these bacteria in the treated wastewater. This will provide additional information necessary for decision-making with respect to risk reduction strategies.

## Declarations

### Author contribution statement

Hlengiwe N. Mtetwa: Conceived and designed the experiments; Performed the experiments; Analyzed and interpreted the data; Wrote the paper.

Isaac D. Amoah, Sheena Kumari, Faizal Bux & Poovendhree Reddy: Conceived and designed the experiments; Contributed reagents, materials, analysis tools or data.

### Funding statement

This work was supported by the 10.13039/501100001322South African Medical Research Council as a sub-grant received from the 10.13039/100000865Bill and Melinda Gates Foundation (96086).

### Data availability statement

Data included in article/supplementary material/referenced in article.

### Declaration of interests statement

The authors declare no conflict of interest.

### Additional information

No additional information is available for this paper.

## Appendix A Supplementary data

Heliyon-supplementary-updated Heliyon-supplementary-updated
