# Supplementary material for: Molecular surveillance of tuberculosis-causing mycobacteria in wastewater
Source: Heliyon. 2022 Feb 4;8(2):e08910. doi: 10.1016/j.heliyon.2022.e08910 (PMC8842018; doi:10.1016/j.heliyon.2022.e08910)
Supplement: Heliyon-supplementary-updated [file mmc1.docx]

Table S1: Target genes and their PCR primer sequences

| **Gene** | **Organism name** | **Primer sequence** | **Reference** |
| --- | --- | --- | --- |
| **16S rRNA gene** | All mycobacterial species | F:5’-GAGATACTCGAGTGGCGAAC-3’  R:5’CAACGCGACAAACCACCTAC-3’ | Chae et al., 2017 |
| **Rv0577** | *M. tuberculosis*complex | F:5’ATGCCCAAGAGAAGCGAATACA-3’  R:5’ AATGTCAGCCGGTTCCGCAA-3’ | Chae et al., 2017 |
| **IS6110** | *M. tuberculosis*complex | F; 5' GGATCCTGCGAGCGTAGGCGTCGG-3’   R; 5' CCTGTCCGGGACCACCCGCGGCAA-3’ | Farah Aldour et al., 2018; Perez-Osorio et al., 2012 |
| **RD8 present** | *M. africanum* | F:5’- GTCGAAGCGGGGCGCTCT -3’  R:5’- GCGCAACGGATTTCCATCGT -3’ | Asante-Poku et al., 2015; Kim et al., 2013 |
| **RD9** | *M. tuberculosis* | F:5’-GTGTAGGTCAGCCCCATCC-3’  R:5’-GTAAGCGCGTGGTGTGGA -3’ | Chae et al., 2017; Perez-Osorio et al., 2012 |
| **RD 4** |  | forward = 5′-ATGTGCGAGCTGAGCGATG-3′ | Hlokwe et al., 2017; Warren et al., 2006 |
|  |  | internal = 5′-TGTACTATGCTGACCCATGCG-3′ |  |
|  |  | reverse = 5′-AAAGGAGCACCATCGTCCAC-3 |  |
| **RD9** |  | forward: 5′-CAAGTTGCCGTTTCGAGCC-3′ | Hlokwe et al., 2017; Warren et al., 2006 |
|  |  | internal: 5′-CAATGTTTGTTGCGCTGC-3′ |  |
|  |  | reverse: 5’GCTACCCTCGACCAAGTGTT-3′ |  |
| **RD12** |  | forward: 5′-GGGAGCCCAGCATTTACCTC-3′ | Hlokwe et al., 2017; Warren et al., 2006 |
|  |  | internal: 5’GTGTTGCGGGAATTACTCGG-3′ |  |
|  |  | reverse: 5′-AGCAGGAGCGGTTGGATATTC-3 |  |
| **RD1** | *M. bovis* | External forward 5′AAGCGGTTGCCGCCGACCGACC | El-Tawab et al., 2016; Warren et al., 2006 |
|  |  | Internal forward 5′CTGGCTATATTCCTGGGCCCGG |  |
|  |  | External reverse 5′GAGGCGATCTGGCGGTTTGGGG |  |
| **RD9** |  | F; 5' ACT CCC AGC GCT CGG CGG TGA CGG TAT CGT 3' | Vasconcellos et al., 2010 |
|  |  | R; 5' ATT CCG TGG GCG CTG CGG CCA ATG TTT GTT 3' |  |
| **RD8 deleted** |  | F-GTCGAAGCGGGGCGCTCT | Kim et al., 2013 |
|  |  | R-GGTTCTTGGCGTCTTGGAAGG |  |
| **RD1** |  | F-CGAGGGGAAGCAGTCCCTGA | Kim et al., 2013 |
|  |  | R-AGGTCGAACTCGCCCGATCC |  |

 Figures S1–S3: Diagrams for the wastewater treatment plant’s configurations chosen for this study


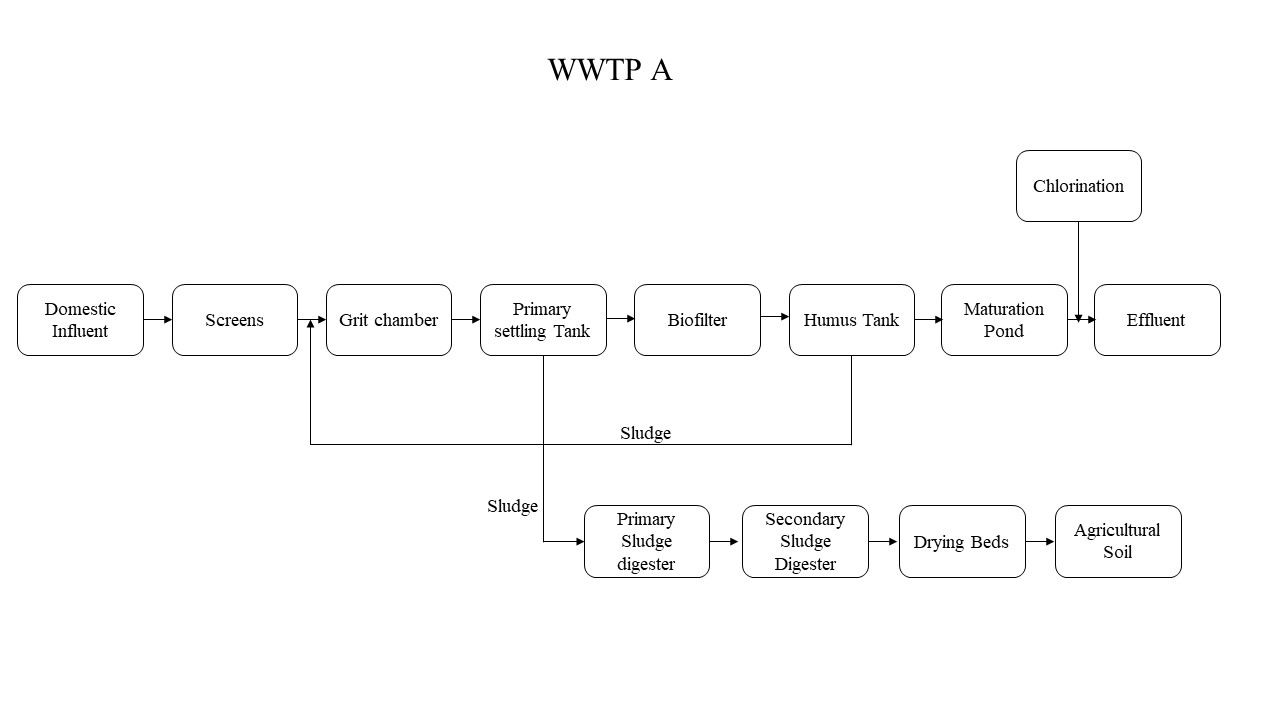


Figure S1: A schematic diagram of WWTP A in Durban, South Africa


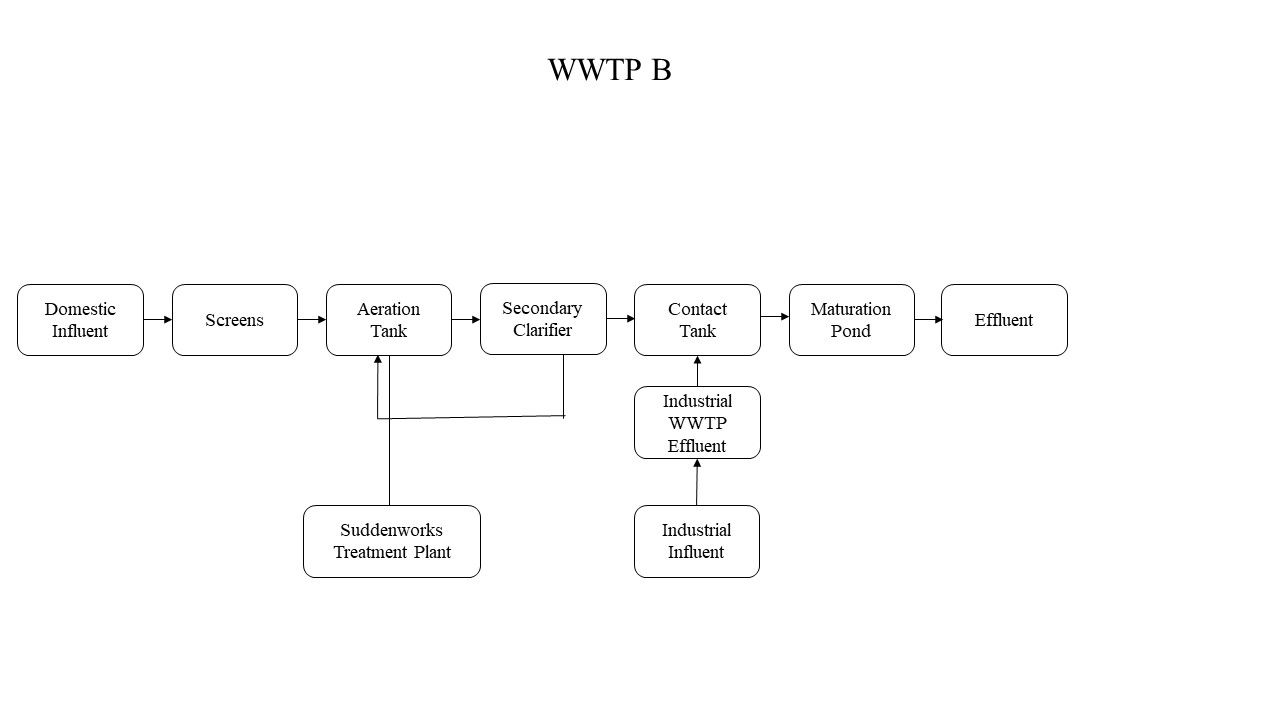


Figure S2: A schematic diagram of WWTP B in Durban, South Africa


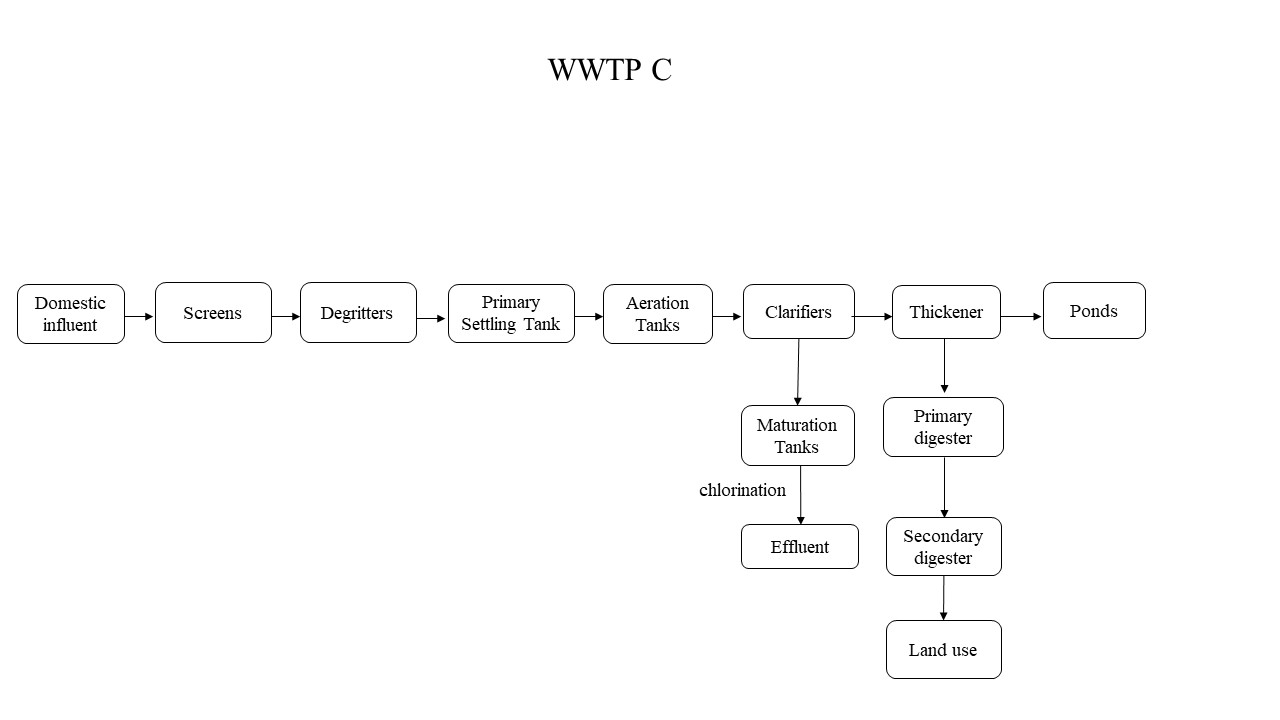


Figure S3: A schematic diagram of WWTP C in Durban, South Africa

## *M. tuberculosis* H37Rv strain limit of detection for the ddPCR assay

The standard/reference *M. tuberculosis* (H37Rv strain) DNA was determined to have an average of 9226(±642.1) copies/mL. The LOD after ten-fold serial dilutions was determined to be 3.0 (±0.06) gc/ml (Figure 2) with an average of 18,892 droplets generated per well.

Figure S4: Limit of detection for *M. tuberculosis* using the droplet digital PCR.

**Key: 0= no dilution (standard/reference strain); -1= 10^-1^ dilution; -2 =10^-2^ dilution; -3 = 10^-3^ dilution; -4=10^-4^ dilution; -5= 10^-5^ dilution; -6=10^-6^ dilution; -7=10^-7^ dilution; -8=10^-8^; -9= 10^-9^ dilution.*

**References**

Farah Aldour, M.S.M., Elhussein, A.R.M., Elkhidir, I.M., Tayeib, S.E., Mohammed Khair, O., Mohamed, N.S., Enan, K.A., 2018. Detection of drug resistant genes of Mycobacterium tuberculosis in Sudanese tuberculosis patients in Khartoum state using multiplex PCR. EC Microbiol. 14, 686–693. <http://repo.nusu.edu.sd/handle/123456789/132>.

Warren, R.M., van Pittius, N.C., Barnard, M., Hesseling, A., Engelke, E., De Kock, M., 2006. Differentiation of Mycobacterium tuberculosis complex by PCR amplification of genomic regions of difference. Int. J. Tubercul. Lung Dis. 10, 818–822.
